# Migration d'une broche dans le canal médullaire cervical après une ostéosynthèse de l'articulation acromio-claviculaire

**DOI:** 10.11604/pamj.2016.24.35.8200

**Published:** 2016-05-10

**Authors:** Bah Aliou, El Alaoui Adil

**Affiliations:** 1 Chirurgie Orthopédique, Centre Hospitalier Métropole Savoie de Chambéry, France; 2 Chirurgie Orthopédique Traumatologique, l'Hôpital Militaire Mohammed V, Rabat, Maroc; 3 Chirurgie Orthopédique et Traumatologique, Centre Hospitalier Universitaire Fèz, Maroc

**Keywords:** Fracture clavicule, embrochage-haubanage, complication ostéosynthèse, Distal clavicle fracture, acromioclavicular joint, osteosynthesis, fracture, pin migration, iatrogenic complication

## Image en médecine

Il s'agit d'un patient de 49 ans, droitier, cuisinier, victime d'un traumatisme de l’épaule gauche au football. Le diagnostic d'une fracture du quart distal de la clavicule gauche était posé. Il bénéficia d'une ostéosynthèse par embrochage-haubanage. Devant des douleurs et une hypoesthésie avec un déficit des interosseux en territoire C8-T1, le bilan radiographique objectivait une migration de broche au niveau du rachis cervical. Elle transperçait le foramen de conjugaison C7-T1 gauche allant au contact du fourreau médullaire mettant en jeu son pronostic vital ou fonctionnel. L'ablation du matériel d'ostéosynthèse en urgence en Neurochirurgie fut réalisée. L’évolution est marquée par une disparition des névralgies et une récupération fonctionnelle acceptable. L'ostéosynthèse par embrochage-haubanage est une des principales techniques chirurgicales des fractures du quart distal de la clavicule ou des disjonctions acromio-claviculaires. Sa principale complication reste la migration de broche.

**Figure 1:**
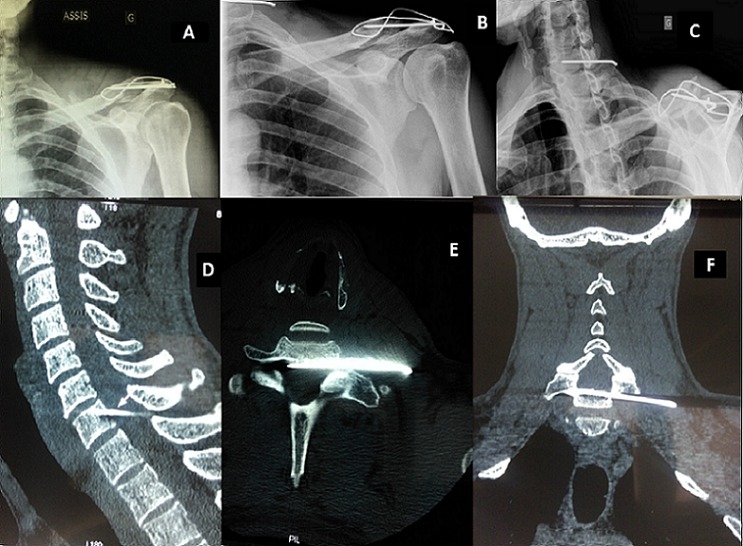
A) radiographie standard de l’épaule gauche face post opératoire montrant l'embrochage- haubanage de l'articulation acromio-claviculaire; B et C) radiographies standard de l’épaule gauche montrant la migration d'une broche d'ostéosynthèse au niveau du rachis cervical; D, E et F) coupes scanographiques (sagittale, axiale et coronale) montrant la broche transperçant le foramen de conjugaison C7-T1 gauche allant au contact du fourreau médullaire

